# Curcumin alleviates persistence of *Acinetobacter baumannii* against colistin

**DOI:** 10.1038/s41598-018-29291-z

**Published:** 2018-07-23

**Authors:** Amanjot Kaur, Prince Sharma, Neena Capalash

**Affiliations:** 1 Department of Biotechnology, Panjab University, Chandigarh, 160014 India; 2 Department of Microbiology, Panjab University, Chandigarh, 160014 India

## Abstract

Persisters are phenotypic variants of normal susceptible bacterial populations that survive prolonged exposure to high doses of antibiotics and are responsible for pertinacious infections and post-treatment relapses. Out of the three antibiotics, *Acinetobacter baumannii* formed the highest percentage of persister cells against rifampicin followed by amikacin and the least against colistin. Colistin-treated cells formed the high levels of reactive oxygen species (ROS) whose quenching with bipyridyl and thiourea led to an increased persister population. Curcumin, a polyphenolic pro-oxidant, significantly decreased persistence against colistin. The quenching of ROS generated by curcumin-colistin combination and the use of resveratrol, an anti-oxidant, with colistin increased the persister population, supporting the significance of ROS in decreased persistence against this combination. The down-regulation of repair genes by this combination in comparison to colistin alone supported the modulation of gene expression in response to ROS and their importance in decreased persistence. Increased membrane permeability by colistin, facilitating the penetration of curcumin into cells and resulting in increased ROS and compromised repair compounded by the decreased efflux of colistin by the inhibition of efflux pumps, may be responsible for enhanced lethality and low persistence. Hence, the curcumin-colistin combination can be another option with anti-persister potential for the control of chronic *A*. *baumannii* infections.

## Introduction

*Acinetobacter baumannii* is a Gram-negative, aerobic pathogen, responsible for nosocomial infections worldwide, including hospital-acquired pneumonia and bloodstream, urinary tract, skin and soft tissue infections^1^. *A*. *baumannii* is one of the six ‘superbugs’ identified by the Infectious Diseases Society of America (IDSA) as the “ESKAPE” group, comprised of *Enterococcus faecium*, *Staphylococcus aureus*, *Klebsiella pneumoniae*, *Acinetobacter baumannii*, *Pseudomonas aeruginosa* and *Enterobacter species*^2^. It figures in the “critical” category of World Health Organisation’s (WHO) priority pathogens list for the development of new antibiotics^3^ and has also been designated as a “red alert” human pathogen characterised as “naturally transformable”, as it can rapidly acquire diverse resistance genes and undergoes genetic modifications conferring resistance to all currently used antibiotics^4^. Apart from multi-drug resistance, *A*. *baumannii* also exhibits multidrug tolerance mediated by persister cells that are responsible for chronic infections^5,6^. These cells show biphasic killing kinetics on treatment with bactericidal antibiotics that kill the majority of the susceptible cells, leaving behind persister cells in the clonal population, which show transitory tolerance to antibiotics^7^. Unlike resistant cells, persister cells do not grow in the presence of antibiotics and arise without undergoing genetic changes. They enter into a state of dormancy, which does not allow the antibiotic to bind to their targets, rendering these cells multidrug tolerant. Having survived the antibiotic stress, these cells re-establish into the same sensitive population, generating the same percentage of persister cells once the stress is removed^8^. Recent studies have reported that the persister phenotype is modulated by starvation, oxidative stress and quorum sensing^9,10^. The enhanced efflux of antibiotics resulting in decreased intracellular antibiotic accumulation also contributes to bacterial persistence^11^.

Current treatment options for infections caused by *A*. *baumannii* are limited. Meropenem, tigecycline, minocycline, amikacin and rifampicin have been used against *A*. *baumannii* infections^12^. Colistin (polymyxin E) and polymyxin B, previously abandoned antibiotics, have now re-emerged as the last-resort and the only effective therapeutic option against multi-drug resistant (MDR) and extreme-drug resistant (XDR) *Acinetobacter* infections^13^. *A*. *baumannii* is reported to form persister cells in response to amikacin and carbenicillin^14^, meropenem^15^, colistin^6^ and polymyxin B^15^. Combination therapy with different antibiotics has been used to combat *A*. *baumannii* infections^16^, but their failure, resulting in a relapse of infections, has necessitated the exploration of combinations of antibiotic with non-antibiotic drugs for enhanced antimicrobial efficacy^17^.

Curcumin (1,7-bis(4-hydroxy-3-methoxyphenyl)-1,6-heptadiene-3,5-dione), a natural polyphenol, is known to exhibit anti-inflammatory, anti-proliferative, antibacterial, anti-biofilm and anti-quorum sensing activities^18^. It is also reported to act in synergism with various antibiotics against Gram-positive and Gram-negative bacteria^18–21^. This is the first study to investigate the anti-persister potential of curcumin in combination with antibiotics, which could be explored as an option to manage recurrent and chronic *A*. *baumannii* infections. In the present study, curcumin significantly decreased the persistence of *A*. *baumannii* against colistin, amikacin and rifampicin. The curcumin-colistin combination was most effective in reducing persistence, which could be due to the increased ROS production and efflux pump inhibition by curcumin aided by the increased membrane permeability by colistin.

## Results

### Antibiotics of different classes induced persister cell formation in *A*. *baumannii*

**Figure 1:**
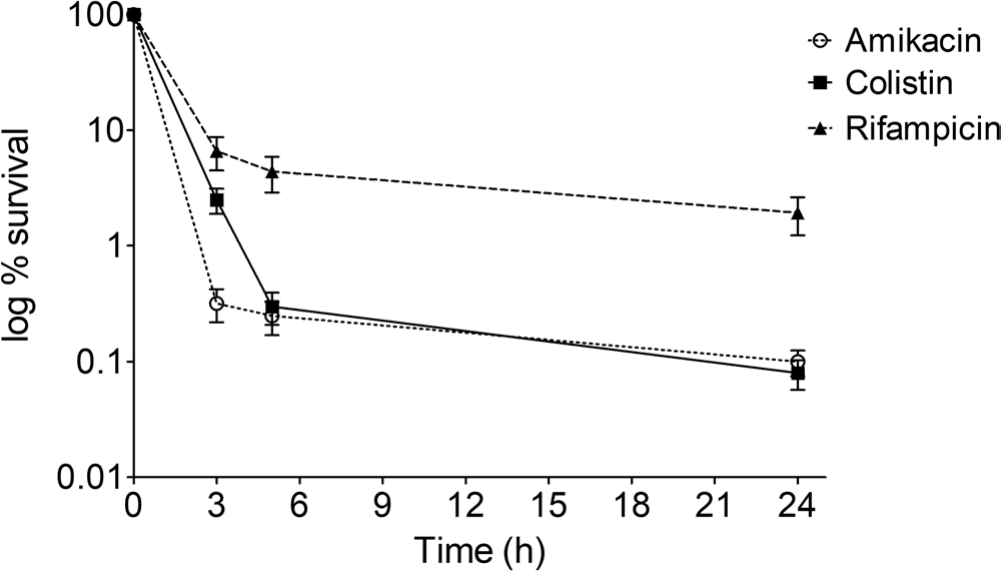
Time dependent persister assay of the late exponential phase cells of *A*. *baumannii* 17978 treated with 40X amikacin, 10X colistin and 20X rifampicin for different time intervals. Untreated cells taken as the control (100% survival) were 1.5 × 10^9^, 1.4 × 10^9^ and 2.1 × 10^9^ CFU/ml, respectively. The data is representative of three independent experiments. Bars represent the mean ± SD.

*A*. *baumannii* 17978 culture in the late exponential phase formed persister cells in response to amikacin and colistin (MIC, 2 µg/ml each) and rifampicin (MIC, 4 µg/ml) at 40X, 10X and 20X MIC, respectively (Suppl. Fig. 1a–c). The time-dependent assay revealed maximum persisters (1.92%) against rifampicin at 20X MIC, 0.10% against amikacin at 40X MIC and the lowest level (0.08%) against colistin (10X MIC) at 24 h; this was revealed by the typical biphasic killing with a sharp decline in the susceptible cells followed by a plateau of the surviving persister subpopulation (Fig. 1). The persister cells were not resistant to these antibiotics, as no change in MIC was observed and there was also no rebounding growth of persister cells. Culture grown from persister cells after each passage was as sensitive to the antibiotic as the parent culture and displayed a biphasic killing pattern, forming a similar proportion of persister cells after each consecutive passage, confirming the non-heritability of persistence (Suppl. Fig. 2).

### Curcumin decreased persistence against antibiotics

**Figure 2:**
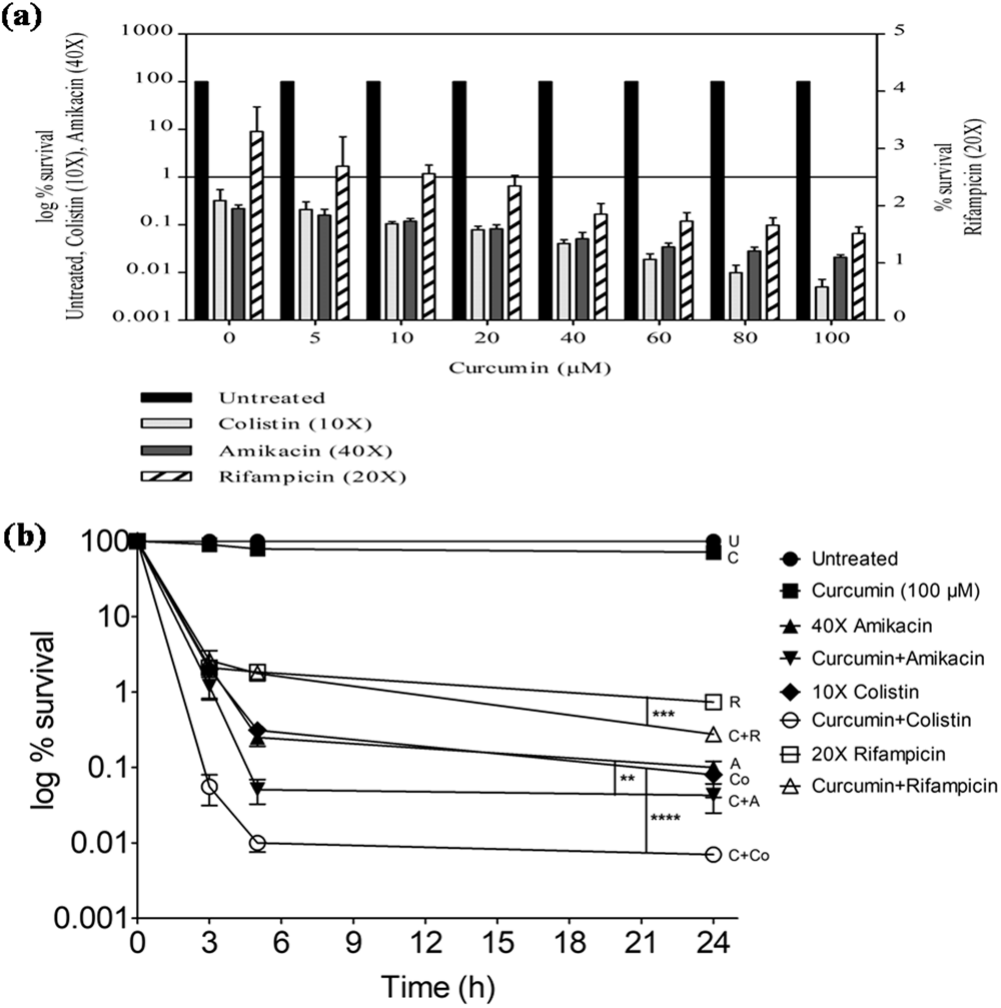
Effect of curcumin on the persister cells formation in the late exponential phase cells of *A*. *baumannii* 17978. (**a**) *A*. *baumannii* cells treated for 5 h with curcumin in combination with 10X colistin, 40X amikacin and 20X rifampicin. Cells treated with 0, 5, 10, 20, 40, 60, 80, 100 µM of curcumin only (100% survival) were 2.18 ± 0.14 × 10^9^ CFU/ml. (**b**) Effect of curcumin (100 µM) on persistence of the late exponential phase cells against 40X amikacin, 10X colistin and 20X rifampicin. Untreated cells taken as the control (100% survival) were 1.2 ± 0.57 × 10^9^ CFU/ml respectively. The data is representative of three independent experiments. Bars represent the mean ± SD. *P ≤ 0.05; **P ≤ 0.01; ***P ≤ 0.001; ****P ≤ 0.0001.

In the presence of 100 µM of curcumin (MIC, 1.2 mM), the treatment of cells in late exponential phase for 5 h (sufficient to obtain persister cells as per time dependent persister assays) with 10X colistin resulted in a 4.30 log-fold (*P* < 0.001) reduction in persister cells formation, followed by a 3.67 log-fold (*P* < 0.01) reduction against 40X amikacin and 1.81 log-fold (*P* < 0.01) reduction against 20X rifampicin (Fig. 2a). The time-dependent persister assay also showed that the combination of 100 µM curcumin with 10X colistin was the most effective (*P* < 0.0001) in decreasing the persistence of *A*. *baumannii* compared to that with amikacin or rifampicin (*P* < 0.01) at 24 h (Fig. 2b). The MDR clinical strain MM6, which formed a high percentage (35.71%) of antibiotic-tolerant cells against 10X colistin (MIC, 1 µg/ml) (Suppl. Fig. 1d), also showed a 3.68 log-fold (*P* < 0.0001) reduction in cells in the presence of curcumin at 24 h (Suppl. Fig. 3). Curcumin killed the pre-formed persister cells too effectively, as the addition of curcumin (100 µM) for 2 h resulted in a 2.03 log-fold decrease in their survival, which reduced to 2.76 log-fold at 24 h (Suppl. Fig. 4).

### Role of reactive oxygen species (ROS) in persistence of *A*. *baumannii* against colistin

**Figure 3:**
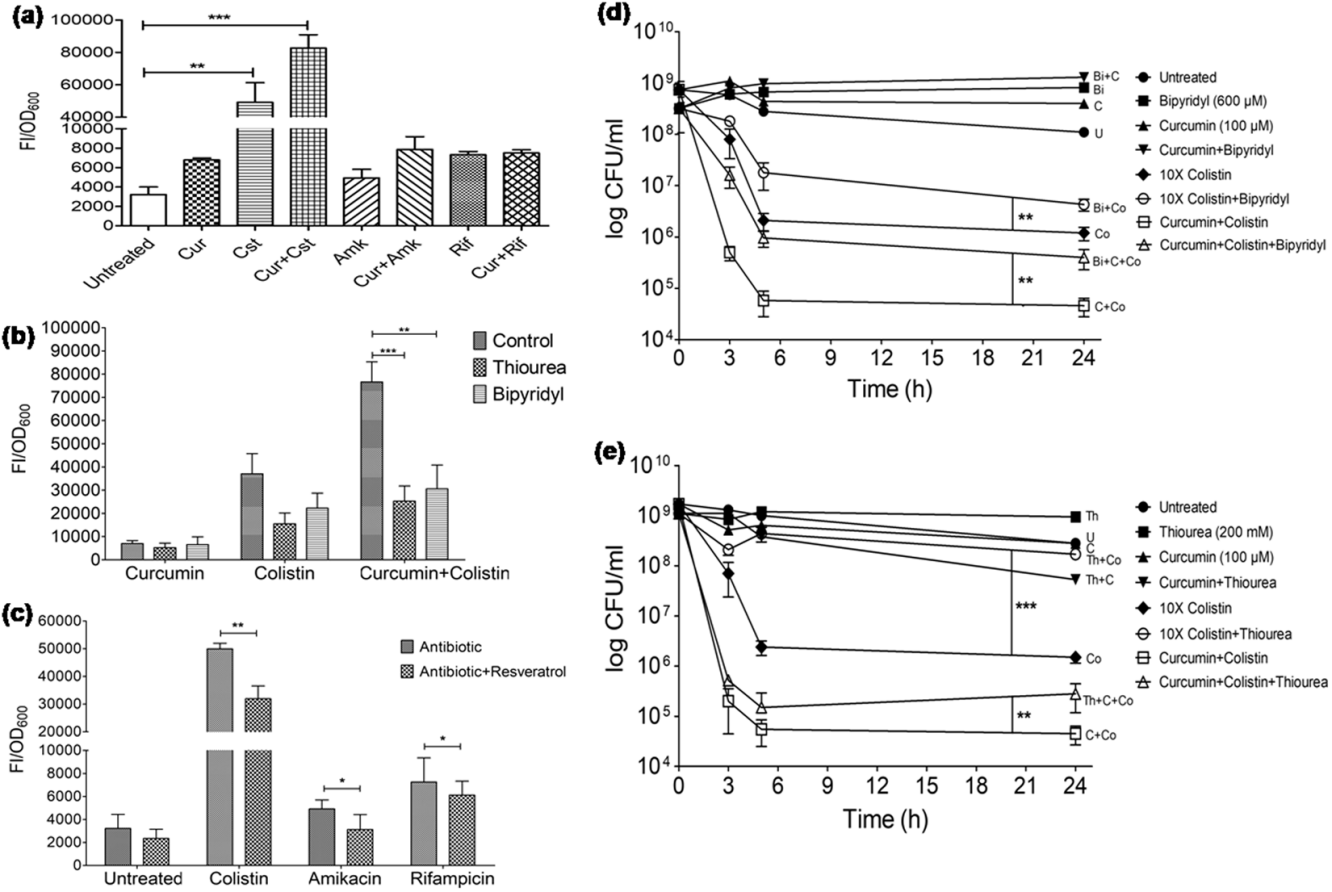
Effect of ROS on the persistence of the late exponential phase cells of *A*. *baumannii* 17978. ROS levels in *A*. *baumannii* cells upon treatment with (**a**) 10X colistin (Cst), 40X amikacin (Amk) or 20X rifampicin (Rif), alone and in combinations with curcumin (Cur; 100 µM); (**b**) curcumin (100 µM), 10X colistin alone and their combination, in presence of thiourea (200 mM) or bipyridyl (600 µM); (**c**) 10X colistin, 40X amikacin and 20X rifampicin, alone and in combinations with antioxidant, resveratrol (100 µM). Persister cells formation in the presence of (**d**) 2,2′-bipyridyl (600 µM), (**e**) thiourea (200 mM) against 10X colistin, 100 µM curcumin and their combination. CFU/ml of untreated cells (100% survival) for (**d**) 7.4 ± 2.8 × 10^8^ and (**e**) 1.7 ± 0.6 × 10^9^. The data is representative of three independent experiments. Bars represent the mean ± SD. *P ≤ 0.05; **P ≤ 0.01; ***P ≤ 0.001.

Antibiotics are known to generate ROS, which adds to their lethality. *A*. *baumannii* cells treated with 10X colistin generated maximum ROS (15.25-fold) (*P* < 0.01) compared to 40X amikacin (1.53-fold) and 20X rifampicin (2.27-fold) (Fig. 3a). ROS generated by 10X colistin decreased to 9.09- (*P* < 0.05) and 6.32-fold (*P* < 0.05) in the presence of sub-inhibitory concentrations of 2,2′-bipyridyl (600 µM) and thiourea (200 mM), respectively (Fig. 3b), with a concomitant increase in the persister cells survival by 8.28-fold (*P* < 0.01) and 2.24 log-fold (*P* < 0.01) at 24 h, respectively (Fig. 3d and e). The addition of curcumin (100 µM) synergistically increased ROS by 25.66-fold (*P* < 0.001) in combination with 10X colistin, while it was 2.44- and 2.33-fold in combination with amikacin and rifampicin, respectively (Fig. 3a). The treatment of *A*. *baumannii* cells with the combination of curcumin and colistin in the presence of sub-inhibitory concentrations of 2,2′-bipyridyl and thiourea decreased ROS to 12.50- (*P* < 0.01) and 10.33-fold (*P* < 0.001), respectively (Fig. 3b), and concomitantly increased the persister cells survival by 20.10-fold (*P* < 0.01) and 7.86-fold (*P* < 0.01) after 24 h, respectively (Fig. 3d and e). Similarly, in MDR strain MM6, 10X colistin in combination with 100 µM curcumin also showed increased ROS production by 13.69-fold (*P* < 0.01) in comparison to 8.17-fold (*P* < 0.01) with colistin alone (Suppl. Fig. 5a). The ROS generated by colistin in combination with curcumin decreased to 9.96-fold (*P* < 0.01) and 7.50-fold (*P* < 0.01) in the presence of 2,2′-bipyridyl and thiourea, respectively (Suppl. Fig. 5b), with a concomitant increase in the survival of antibiotic-tolerant cells by 4.35-fold and 1.76-fold at 24 h, respectively (Suppl. Fig. 5c,d). Resveratrol (MIC, 600 µM) which showed antioxidant activity at 100 µM by decreasing ROS generated by colistin from 15.5-fold to 9.9-fold (*P* < 0.01) (Fig. 3c) also increased persistence by 19.04 fold against 10X colistin at 24 h (Suppl. Fig. 6) supporting the significance of ROS in persistence.

### Expression of antioxidant enzymes, repair genes and isocitrate lyase

**Figure 4:**
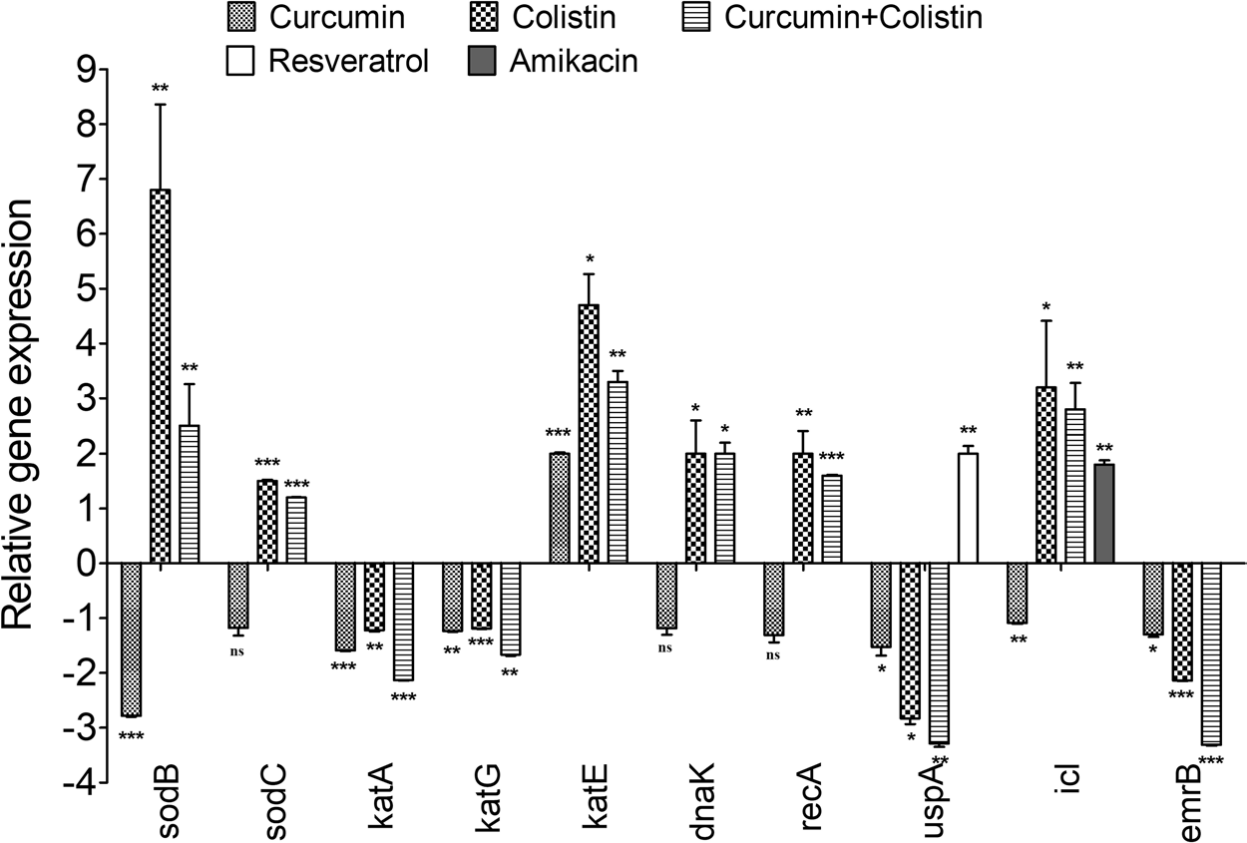
Relative expression of genes involved in the oxidative stress response [superoxide dismutases *sodB*, *sodC*; catalases *katA*, *katE*, *katG*], repair [*recA*, *dnaK*, *uspA*], isocitrate lyase *icl* and *emrB* of MFS efflux pump EmrAB on treatment with curcumin or resveratrol (100 µM), amikacin (40X), colistin (10X) and colistin-curcumin combination in the late exponential phase cells of *A*. *baumannii* 17978. Untreated cells was taken as the control with basal level expression indicated as 1. The data is representative of three independent experiments. Bars represent the mean ± SD. *P ≤ 0.05; **P ≤ 0.01; ***P ≤ 0.001; ns as non-significant.

A significant up-regulation of 6.8-fold in the expression of *sodB* was observed with 10X colistin which dropped to 2.5-fold in *A*. *baumannii* cells exposed to the combination of colistin (10X) and curcumin (100 µM). This change in *sodB* expression might be due to curcumin, which was found to down-regulate this by 2.8-fold. However, slight up-regulation was observed in *sodC* levels on exposure to colistin and its combination with curcumin. A marginal decrease in *katA* and *katG* levels by 1.2-fold was observed with colistin, which further decreased significantly by 2.12-fold and 1.66-fold, respectively, following treatment with curcumin-colistin combination. Conversely, a significant increase (4.7-fold) was observed in *katE* expression with colistin treatment, which dropped to 3.3-fold in cells treated with a colistin and curcumin combination (Fig. 4). On the whole, curcumin was found to decrease the expression of stress response genes, with an exception in *katE* expression, which was up-regulated by 2.05-fold.

A significant up-regulation in *recA* and *dnaK* levels by 2 fold was observed with colistin, which dropped to 1.65-fold for *recA*, while it remained the same for *dnaK* on exposure to the combination of colistin and curcumin. However, curcumin alone showed only a marginal down-regulation of *recA* and *dnaK* levels. A significant down-regulation of 3-fold in *uspA* levels was observed with colistin treatment, which further decreased to 3.35-fold with the colistin and curcumin combination. Curcumin alone also down-regulated the expression of *uspA* by 1.52-fold (Fig. 4). However, resveratrol (100 µM) as an antioxidant was found to increase *uspA* expression by 2-fold (Fig. 4).

A significant up-regulation in *icl* levels by 3.2-fold was observed with colistin, which dropped to 2.8-fold with the combination of colistin and curcumin (Fig. 4). However, curcumin alone marginally decreased *icl* levels and amikacin was found to up-regulate it by 1.80-fold (Fig. 4).

### Change in membrane permeability

**Figure 5:**
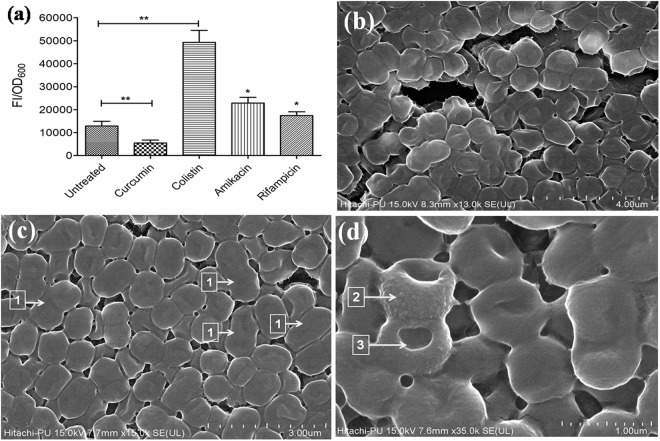
(**a**) Effect of curcumin (100 µM), colistin (10X), amikacin (40X) and rifampicin (20X) on the outer membrane permeability of the late exponential phase cells of *A*. *baumannii* 17978; detected by NPN assay. The data is representative of three independent experiments. Bars represent the mean ± SD. *P ≤ 0.05; **P ≤ 0.01; ***P ≤ 0.001. Scanning Electron Microscope images of *A*. *baumannii* 17978 cells (**b**) without any treatment showing smooth cell surface and intact membrane; (**c**) after exposure for 1 h to curcumin (100 µM) showing elongated cells (arrow 1); (**d**) after treatment with 10X colistin for 1 h showing surface roughness (arrow 2) and disruption in membrane integrity with visible pore (arrow 3).

The treatment of late exponential phase cells with 10X colistin showed increased membrane permeability as there was a significant (3.83-fold, *P* < 0.01) increase in NPN fluorescence compared to untreated cells, while there was no enhancement in fluorescence with curcumin. Treatment with 40X amikacin showed a 1.78-fold (*P* < 0.05) increase in fluorescence, whereas with 20X rifampicin it was 1.35-fold (*P* < 0.05) (Fig. 5a).

### Morphology of colistin and curcumin-treated cells

Field emission scanning electron microscopy (FESEM) revealed that cells treated with curcumin displayed clear and smooth membrane surfaces with no perturbation in the membrane structure, however cells were elongated (Fig. 5c) compared to the untreated cells (Fig. 5b). Colistin treatment showed surface roughness and disruption in membrane integrity with visible pores (Fig. 5d) which increased with exposure time.

### Curcumin as an efflux pump inhibitor (EPI)

#### Change in MIC

The checkerboard assay of curcumin in combination with colistin against *A*. *baumannii* resulted in a fractional inhibitory concentration index (FICI) of 0.29, representing a synergistic interaction. Curcumin at 100 µM effectively reduced the MIC of colistin from 2 to 0.5 µg/ml against *A*. *baumannii*, as represented by a modulation factor (MF) of 4, and that of amikacin from 2 to 1 µg/ml with an MF of 2. However, there was no change in the MIC of rifampicin.

#### Accumulation of EtBr (ethidium bromide)

**Figure 6:**
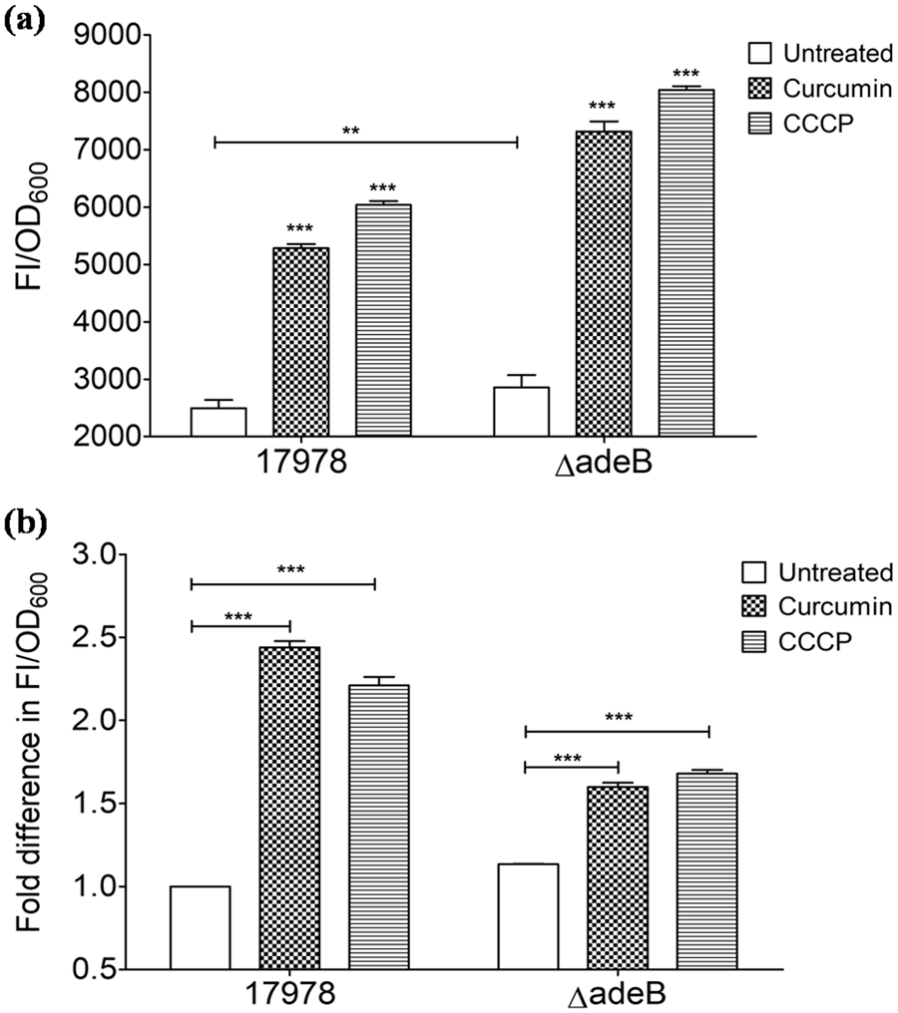
Ethidium bromide accumulation (**a**) and efflux (**b**) in *A*. *baumannii* 17978 and its efflux pump mutant strain (∆*adeB*) in the presence of 100 µM curcumin or CCCP (positive control) after 60 s of exposure. The data is representative of three independent experiments. Bars represent the mean ± SD. *P ≤ 0.05; **P ≤ 0.01; ***P ≤ 0.001.

The addition of curcumin to *A*. *baumannii* cells significantly (*P* < 0.001) enhanced EtBr accumulation (2.11-fold) after 60 sec of exposure, similar to carbonyl cyanide 3-chlorophenylhydrazone (CCCP)-treated cells (2.41-fold). The efflux pump mutant strain (∆*adeB*) deficient in the AdeB transporter of AdeABC efflux pump accumulated higher levels of EtBr compared to the parental strain by 1.14-fold (*P* < 0.01), which showed pronounced accumulation (*P* < 0.001) in the presence of curcumin (2.93-fold) and CCCP (3.22-fold) (Fig. 6a).

#### Efflux of EtBr

Curcumin was found to significantly (*P* < 0.001) inhibit the efflux of EtBr in *A*. *baumannii* as shown by the 2.44-fold increased fluorescence similar to that observed with CCCP (2.21-fold). Significant (1.60-fold, *P* < 0.001) inhibition of EtBr efflux was also observed in *A*. *baumannii* ∆*adeB* by curcumin, while it was 1.68-fold with CCCP compared to untreated ∆*adeB* cells (Fig. 6b).

#### Expression of *emrB* gene of EmrAB efflux pump

A significant down-regulation (2.1-fold) was observed in e*mrB* levels on treatment with colistin, which further decreased (3.3-fold) on treatment with colistin-curcumin combination. Curcumin alone also decreased *emrB* levels by 1.3-fold (Fig. 4).

## Discussion

This study has demonstrated that *A*. *baumannii* forms varying levels of persister cells, which are lowest with colistin, higher with amikacin and the highest with rifampicin. This variation was also strain- and growth phase-dependent, as more persister cells were formed in the stationary than in the log phase (Suppl. Table S1). The variation in persister fraction was also due to differences in the modes of action of antibiotics^22^. *A*. *baumannii* strains from bloodstream infections have shown variation in persister formation against colistin^6^, and growth phase-related variation has also been reported in *E*.*coli*^23^ and *Burkholderia pseudomallei* Bp8^24^. Antibiotics also act by generating ROS, causing a disturbance in metabolism and respiration^25^. Polymyxins lead to the rapid death of *A*. *baumannii*, *E*. *coli* and *Francisella novicida* due to the oxidative damage to DNA, proteins and lipids^26^.

Curcumin is reported to possess both anti-oxidant and pro-oxidant properties^27^. Curcumin at 100 µM acted as a pro-oxidant and aggravated ROS production synergistically in combination with colistin, thus significantly reducing the survival of persister cells. Increased persister cells survival in the presence of ROS quenchers *viz*. 2,2′-bipyridyl, an iron chelator and a potent inhibitor of Fenton reaction, thiourea, a hydroxyl radical scavenger, and resveratrol, a natural phenol known to exhibit anti-oxidant properties^28^, underlined the importance of ROS in modulating the persistence of *A*. *baumannii*.

Although up-regulated by colistin, the oxidative stress response genes *viz*. Fe-Mn superoxide dismutase (*sodB*) and monofunctional catalase (*katE*) showed relatively decreased expression following treatment with the combination of colistin and curcumin. This reduced expression, in addition to the down-regulation of monofunctional (*katA*) and bi-functional (*katG*) catalase, disarmed the protective response of the organism to oxidative stress generated by the combination of colistin and curcumin through ROS, which might be responsible for its enhanced lethality in comparison to colistin alone. *dnaK* encoding a chaperone was important in the maintenance of persister cells in *E*. *coli*^29^ and *S*. *aureus*^30^. However, it did not seem to be important for persister cell survival in *A*. *baumannii*, as there was no change in its expression following exposure to colistin and curcumin. However, there was a significant decrease in the expression of universal stress protein (*uspA*), which has been shown to play an important role in oxidative stress defence and for H_2_O_2_ resistance in *E*. *coli*^31^. In contrast to curcumin (pro-oxidant), resveratrol, an anti-oxidant, significantly up-regulated UspA levels and enhanced persister survival. Hence, the modulation of *uspA* expression by ROS and its maximum down-regulation with the colistin-curcumin combination suggested its importance in persister cells survival, which needs further confirmation by *uspA* knockout in *A*. *baumannii*.

Glyoxylate shunt is induced during oxidative stress in *P*. *aeruginosa*^32,33^ and *B*. *pseudomallei*^34^; isocitrate lyase (ICL) is the first enzyme of this shunt^32^. Reduced persisters against tobramycin on the inhibition of ICL in *B*. *cepacia* biofilm cells also projected it as an important persistence factor^35^. *icl* expression in *A*. *baumannii* was also significantly up-regulated on exposure to high ROS generating combination of colistin and curcumin. However, the up-regulation of *icl* was not sufficient enough to enhance persister cells survival in *A*. *baumannii*. Hence, the involvement of *icl* in the persistence in *A*. *baumannii* needs further exploration.

The outer membrane of Gram-negative bacteria is the first line of defence against lethal compounds. Curcumin, at a concentration of 100 µM, has been shown to damage the membranes of Gram-positive and Gram-negative bacteria^36^. However, *A*. *baumannii* cells treated with sub-inhibitory concentration of curcumin (100 µM) did not show any change in the membrane permeability, while colistin showed a significant increase. Colistin is known to cause membrane permeabilisation in pan-drug resistant Gram-negative bacteria^37^. It interacts electrostatically with the outer membrane of Gram-negative bacteria and competitively displaces Mg^2+^ and Ca^2+^ divalent cations that stabilise the lipopolysaccharide layer, thus disrupting the membrane integrity^38^. This might have led to the better penetration of curcumin in colistin-treated cells and subsequently increased levels of ROS and low persister cells viability. Electron micrographs of *A*. *baumannii* cells also showed disruptions of the membrane integrity by colistin and not by curcumin; however, some of the cells displayed increased cell length. Curcumin has been shown to inhibit cell division resulting in increased cell length in *Bacillus subtilis* also^39^.

Bacterial persisters adopt a two-pronged strategy to survive antibiotic attack by slowing down most of the physiological processes and simultaneously activating their efflux systems to remove intracellular antibiotics, leading to tolerance^11^. Efflux pump inhibitors (EPIs) such as NMP and PAβN act synergistically with antibiotics by blocking the efflux pumps, resulting in their increased efficacy through intracellular accumulation and decreased persister formation^11^. Curcumin increased EtBr accumulation in *A*. *baumannii*, comparable to CCCP, an EPI that dissipates proton motive force (PMF). ∆*adeB* strain with ineffective AdeABC efflux pump showed the increased accumulation of EtBr. Further enhancement in accumulation on the addition of curcumin or CCCP suggested that curcumin, like CCCP, was also inhibiting other efflux pumps.

Curcumin has been shown to exhibit EPI activity at 135 µM against carbenicillin, ceftazidime, meropenem, ciprofloxacin and gentamicin by decreasing their MICs in *P*. *aeruginosa* strains^40,41^ and also acted as an inhibitor of the NorA multidrug efflux pump in *S*. *aureus*, even at 25 µM^42^. In *A*. *baumannii*, there was also a 4-fold reduction in the MIC of colistin; this may help in a reduction of the dose of colistin which is nephrotoxic. A significant decrease in *emrB* expression on treatment with colistin-curcumin combination may be responsible for reduction in MIC. The involvement of EmrAB efflux pump belonging to Major Facilitator Superfamily (MFS) in colistin susceptibility has been reported in *A*. *baumannii*^43^. The curcumin-polymyxin B combination has been found to be effective against *A*. *baumannii*, *P*. *aeruginosa* and *S*. *aureus* associated with traumatic wound infections^21^. The AdeABC efflux pump has been shown to be responsible for the efflux of aminoglycosides in *A*. *baumannii*^44^. Hence, the reduction in the MIC of amikacin could be due to the inhibition of this pump by curcumin. However, no change in the MIC of rifampicin was observed in the presence of curcumin and their combination was also less effective in decreasing persister cells survival in comparison to that with colistin and amikacin. It is possible that curcumin did not inhibit the AdeIJK efflux pump in *A*. *baumannii*, specific for rifampin efflux^45^. Hence, curcumin at sub-MIC concentration fulfilled the criteria for EPIs^46^.

**Figure 7:**
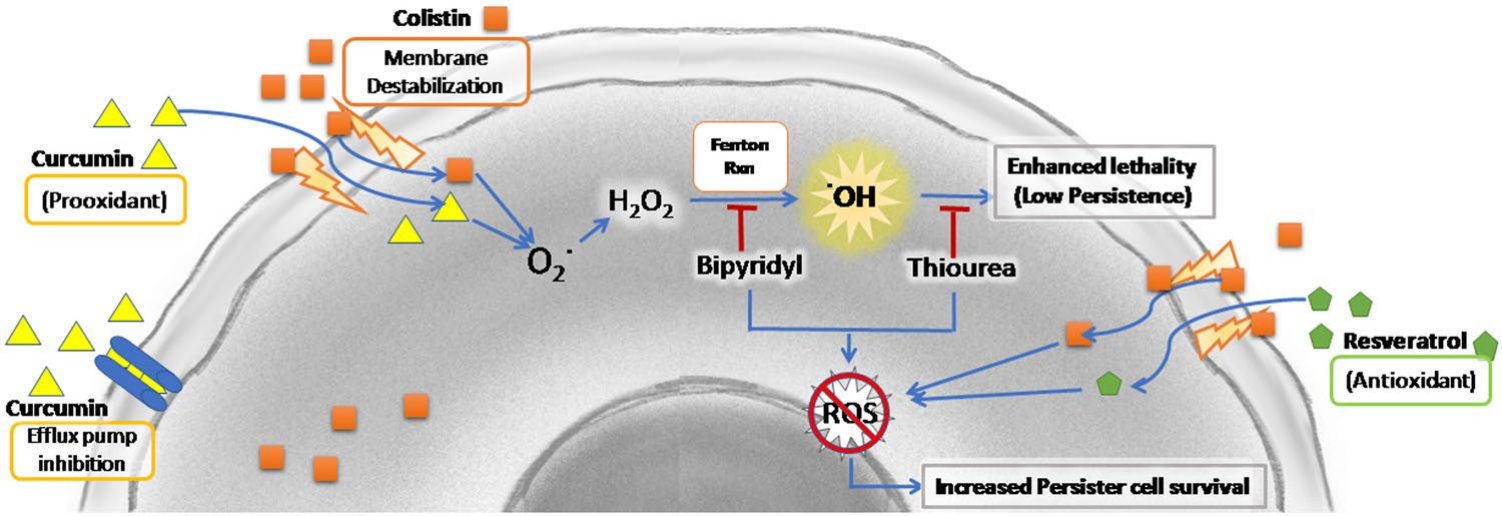
Proposed mechanism involved in decreasing the persistence of *A*. *baumannii* against colistin in combination with curcumin. Increased membrane permeability by colistin facilitated the penetration of curcumin into the cells resulting in increased ROS and compromised repair. Further the decreased efflux of colistin by curcumin may be responsible for the enhanced lethality and low persistence.

The present study shows that curcumin at sub-inhibitory concentration can enhance the therapeutic efficacy of antibiotics. Damage caused to the outer membrane of *A*. *baumannii* by colistin, facilitating the penetration of curcumin, resulted in increased ROS and compromised oxidative stress defence. The decreased efflux of colistin by curcumin, may be responsible for the enhanced lethality and low persistence (Fig. 7) in the colistin-curcumin combination.

## Materials and Methods

### Bacterial strains

*Acinetobacter baumannii* strains used in the study were routinely maintained and grown in Luria-Bertani (LB) broth at 37 °C under shaking conditions. The ATCC 17978 strain was procured from the American Type Culture Collection (ATCC, Manassas, VA, USA). *A*. *baumannii* MM6, a multidrug resistant clinical strain, was isolated from endoscopic tracheal secretion and was sensitive to only colistin and meropenem. The efflux pump mutant strain *A*. *baumannii* ∆*adeB* ATCC 17978 was obtained as gift from Maria Tomas from the Department of Microbiology, Complejo Hospitalario Universitario A Coruña-INIBIC, La Coruña, Spain^47^.

### Determination of MICs

MICs of amikacin, colistin, rifampicin, curcumin and resveratrol against *A*. *baumannii* were determined by the broth microdilution method according to CLSI guidelines^48^, with antibiotics serially diluted in a range from 0.25 to 128 µg/ml and compounds from 0.25 µM to 2 mM. The concentration with no visible growth was taken as the MIC.

### Persister assay

*A*. *baumannii* strains were cultured at 37 °C for 16 h in LB, diluted 1:100 in fresh medium, and incubated until the late exponential phase (4 h); these were treated with either different concentrations of antibiotics (X MIC) for 3 h for the dose-dependent persister assay, or with fixed concentrations of antibiotics (20 µg/ml colistin/80 µg/ml amikacin or rifampicin), at which persister cells were formed for different (3 to 24 h) time intervals for the time-dependent persister assay. Cells after treatment were harvested by centrifugation at 8000 × g for 10 min, washed and diluted serially in 10 mM phosphate buffer saline (PBS), pH 7.2; then, a 10 µl aliquot of each dilution was spotted onto LB-agar to determine the colony-forming units per ml (CFU/ml). Only dilutions that yielded 10–100 colonies were counted. Percentage survival was determined by dividing the CFU/ml of the treated sample with that of the untreated sample^49^. To determine the development of resistant cells, the treated population was spotted on LB agar containing the respective antibiotics (20 µg/ml colistin/80 µg/ml amikacin or rifampicin). The persister cells harvested after 24 h were also checked for any change in MICs.

For a dose-dependent persister assay in the presence of curcumin, the late exponential phase cells of *A*. *baumannii* 17978 were exposed for 5 h to antibiotics (10X colistin, 40X amikacin and 20X rifampicin) in the presence of different sub-MIC concentrations (5 to 100 µM) of curcumin; for a time-dependent persister assay, late exponential phase cells were exposed to the respective antibiotics in the presence of 100 µM curcumin or resveratrol for different time intervals. 200 mM thiourea and 600 µM 2,2′-bipyridyl were added to the cells to quench ROS during treatment with 10X colistin with or without 100 µM curcumin for the time-dependent persister assay. Late exponential phase cells of *A*. *baumannii* MM6 were also exposed to 10X colistin in the presence of 100 µM curcumin for different time intervals to determine time-dependent persistence.

To determine whether curcumin accelerated the cell death of the pre-formed persisters against colistin, 100 µM curcumin was added to the persister cells formed after treatment of *A*. *baumannii* 17978 late exponential phase cells with 10X colistin for 6 h. The effect of curcumin on pre-formed persister cells was observed up to 24 h and CFU/ml at each time point were compared against the CFU/ml of the fraction exposed to 10X colistin only at the respective time interval.

### Non-heritability assay

Persister cells surviving the antibiotic treatment (10X colistin, 40X amikacin or 20X rifampicin) up to 24 h were harvested, washed with PBS and regrown for 24 h at 37 °C in LB broth without antibiotics. The culture was diluted 1:100 with fresh LB broth to obtain late exponential phase cells and again treated with the respective antibiotics up to 24 h. This cycle was repeated three times and antibiotic-treated cells from each passage were withdrawn at different time intervals (0, 3, 5, 24 h), to determine the percentage survival of persister cells^14^.

### ROS estimation

Late exponential phase cells were harvested, washed and re-suspended to 10^7^ CFU/ml in PBS. The cell suspension (800 µl) was treated with antibiotics alone (10X colistin, 40X amikacin or 20X rifampicin) or 100 µM curcumin or resveratrol, or the combination of these for 1 h at 37 °C and 180 rpm. Five µM of 2′,7′-dichlorofluorescin diacetate (DCFDA) was added to the cell suspension and incubated at 37 °C for 30 min in the dark; fluorescence was determined using a multimode microplate reader (BioTek) with excitation and emission wavelengths of 488 and 530 nm, respectively^50^. The fluorescence intensity (FI)/OD_600_ was calculated for normalising the fluorescence with respect to growth. Untreated cells were processed similarly and used as the control.

For ROS quenching experiments, the late exponential phase cells were treated with antibiotics in the presence of quenchers *viz*. 200 mM thiourea or 600 µM 2,2′-bipyridyl, and processed similarly to quantitate ROS as explained above.

### Outer membrane permeabilisation assay using N-phenyl-1-napthylamine dye

Late exponential phase cells (1.0 ml) were pre-treated for 1 h at 37 °C with 100 µM curcumin, 10X colistin, 40X amikacin or 20X rifampicin. Cells were harvested, washed and re-suspended in 1.0 ml PBS. Fluorescence was measured in Genetix 96-well black microtiter plate containing 200 µl of the bacterial cell suspension and 10 µM NPN, immediately at excitation and emission wavelengths set at 350 and 420 nm, respectively. Fluorescence was normalised with growth (OD_600_) of the respective samples^51^. Untreated cells were taken as the control.

### Scanning electron microscopy

FESEM was used to evaluate the morphological changes in *A*. *baumannii* 17978 cells after treatment with curcumin or colistin. For SEM sample preparation, *A*. *baumannii* 17978 late exponential phase cells were exposed to 100 µM curcumin and 20 µg/ml colistin (10X) for 1 h and 3 h and centrifuged at 1400 × g for 5 min. The cells were washed with PBS (pH 7.4) twice and fixed overnight at 4 °C with 2.5% glutaraldehyde. Cells were washed thrice with PBS (pH 7.4) and dehydrated in a series of graded ethanol (30–100%). The untreated control cells were also processed similarly. The specimens were finally suspended in 70% ethanol and air-dried, coated with gold particles via ion sputter (MC 1000, HITACHI) and examined using HITACHI SU8010 FE-SEM^52^.

### Synergistic interaction studies

The checkerboard assay to determine the synergistic activity of curcumin with colistin against *A*. *baumannii* 17978 was performed in a 96-well plate with curcumin diluted along the x-axis and colistin along the y-axis^53^. The range of concentrations for each agent was five dilutions lower and two dilutions higher than the MIC; the interaction between the two agents was calculated by the fractional inhibitory concentration index (FICI) of the combination. The FIC was calculated as the complete inhibition of bacterial growth in combination. The FICI was calculated according to the equation: FICI = FIC_Curcumin_ (MIC_Curcumin_ in combination/MIC_Curcumin_ alone) + FIC_Colistin_ (MIC_Colistin_ in combination/MIC_Colistin_ alone). A FICI value ≤ 0.5 indicated synergy, >0.5 and ≤4.0 indicated indifference and >4.0 indicated antagonism.

### MIC reversal

To determine the ability of curcumin to potentiate the effect of colistin, amikacin and rifampicin, *A*. *baumannii* 17978 was grown in the presence of 100 µM curcumin and varying sub-MIC concentrations of the antibiotics in LB broth for 16 h at 37 °C. The ability of curcumin to reverse the MIC of antibiotics was determined as a fold-reduction in MIC and represented as the Modulation factor (MF) calculated as MIC_Drug_/MIC_Drug_ in combination^54^.

### Ethidium bromide accumulation assay

Late exponential phase cells (5.0 ml) were harvested and re-suspended in 5.0 ml PBS (pH 7.4) containing 0.4% glucose. The cells (200 µl) were transferred to Genetix 96-well black microtiter plate, before curcumin or CCCP (100 µM each) was added. Thereafter, EtBr was added (2 µg/ml) and EtBr fluorescence was measured for 60 s with excitation and emission wavelengths of 544 and 590 nm, respectively^55^. Fluorescence was normalised with growth (OD_600_) of the respective samples.

### Ethidium bromide efflux assay

Late exponential phase cells (800 µl) were harvested, washed and re-suspended in PBS and treated with 100 µM of curcumin or CCCP (positive control) along with EtBr (2 µg/ml) and glucose (0.4%). The untreated cell suspension was similarly processed and used as control. The samples were incubated at 37 °C for 1 h for maximal uptake of EtBr, harvested, washed twice to remove any residual EtBr and re-suspended in PBS. The EtBr efflux was determined by measuring the EtBr fluorescence for 60 s with excitation and emission wavelengths of 544 and 590 nm, respectively, and was normalised to EtBr fluorescence of untreated cells^54^.

### Quantitative PCR

Total RNA was isolated by TRI Reagent (Sigma) from 1.0 ml of late exponential phase cells of *A*. *baumannii* 17978 treated with curcumin or resveratrol (100 µM), amikacin (40X), colistin (10X) and colistin-curcumin combination for 30 min. In order to avoid the isolation of RNA from lysed cells, colistin-treated cells were initially centrifuged at 1000 × g for 8 min and the supernatant was decanted carefully to remove ghost cells and debris from the sample^56^. The cell pellet was washed twice with PBS (pH 7.4) containing 10X colistin and centrifuged at 1000 × g for 5 min to obtain unlysed cells. RNA was converted to cDNA using the Verso cDNA kit (Thermo Scientific). The expression of genes involved in the oxidative stress response (superoxide dismutases *sodB*, *sodC*; catalases *katA*, *katE*; catalase-peroxidase *katG*), repair (*recA*, *dnaK*, *uspA*), isocitrate lyase (*icl*) and for the *emrB* of the MFS efflux pump EmrAB, was assessed using SYBR green mastermix (Thermo Scientific) and the Eppendorf Realplex System. Primers for qRT-PCR were designed using the Primer 3 software (Table 1) with 16S rRNA as the housekeeping gene. Three biological replicates and two technical replicates were performed for each gene tested and the relative expression of genes was calculated by the 2^−∆∆ct^ method^57^.

**Table 1:** List of primers used for gene expression analysis by qRT-PCR.

Gene	Primer Sequence	
*16S*	Fwd	GAGGAAGGTGGGGATGACGT
	Rev	AGGCCCGGGAACGTATTCAC
*sodB*	Fwd	GGTTGGGCTTGGTTAGTTGCTG
	Rev	GTTGGCCTGCAAGTTTTGCATT
*sodC*	Fwd	ACAGCCCATGCACTTACCAC
	Rev	GCAGGGGCACAAGATGGATT
*katA*	Fwd	ACCTGATCCAGCCCTTGTTG
	Rev	TGAGGACGCATAGACCAACG
*katG*	Fwd	ATTTCTTCATCATCCATTGCC
	Rev	GGCGATGAAAAAGAATGGTTA
*katE*	Fwd	GACTTCGATTTGCTGGACCC
	Rev	GTCGGGCTTGTAAAAGTGGG
*recA*	Fwd	CACGCCCTAGACCCTCAATA
	Rev	AGTCACCCATCTCACCTTCG
*dnaK*	Fwd	GAAATCGCGGACCTTGATGG
	Rev	GACCAGTCGCATCAGCAGTGA
*uspA*	Fwd	TTCTTTGGCAGCAGCACGAC
	Rev	CACCTTCTTCAGCGCATAGGG
*icl*	Fwd	AGGCACTCAGATTGACCGTG
	Rev	TCGTACGCTTGTTGACGGAA
*emrB*	Fwd	GCGGGATGATTTCCCGACTTC
	Rev	TGAGCGTTTTGGTTCTGGAAA

## Electronic supplementary material

Supplementary data
