# Supplementary material for: Curcumin alleviates persistence of Acinetobacter baumannii against colistin
Source: Sci Rep. 2018 Jul 23;8:11029. doi: 10.1038/s41598-018-29291-z (PMC6056455; doi:10.1038/s41598-018-29291-z)
Supplement: Supplementary file 1 — Supplementary data [file 41598_2018_29291_MOESM1_ESM.pdf]

Supplementary Data:

Curcumin alleviates persistence of *Acinetobacter baumannii* against colistin

Amanjot Kaur[1], Prince Sharma[2], Neena Capalash[1]*

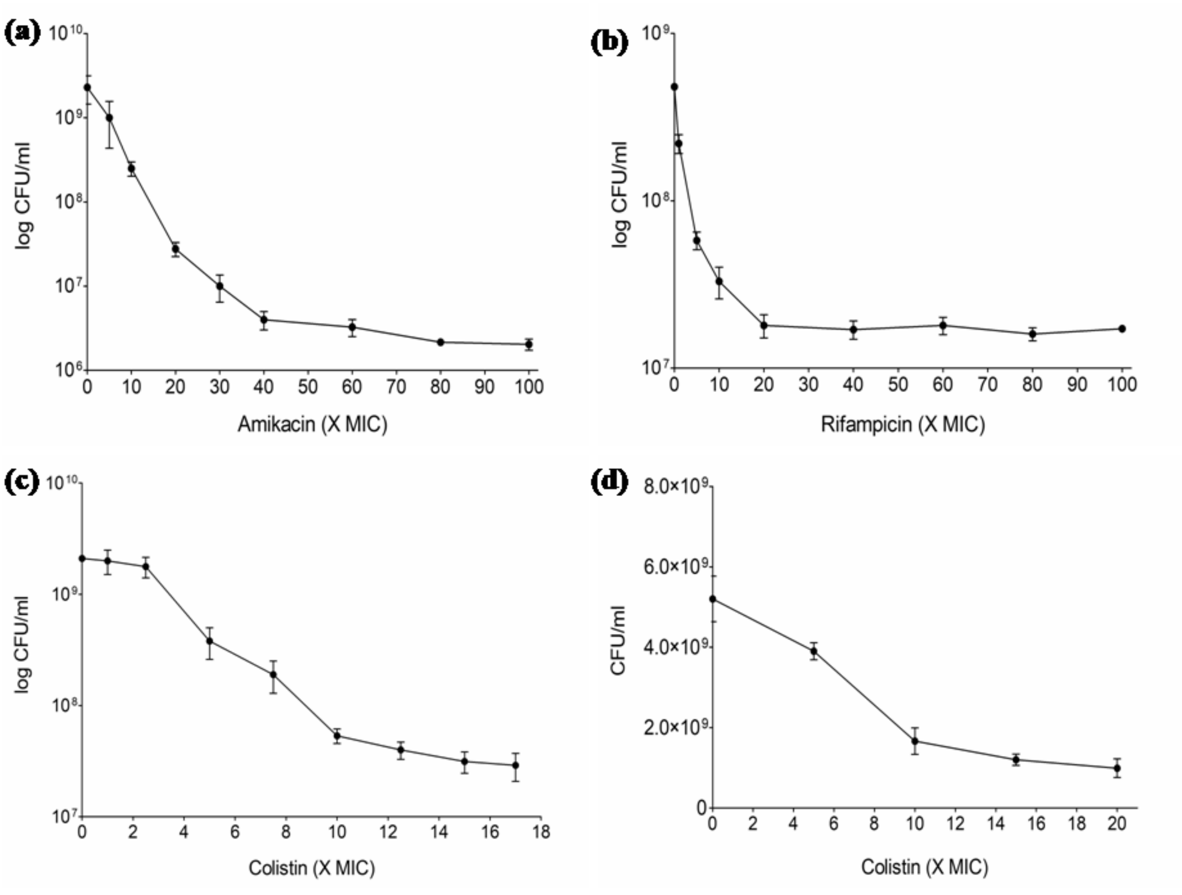

Suppl. Fig. 1: Concentration-dependent persister assay. Effect of (a) amikacin, (b) rifampicin and (c) colistin on persister cell formation in the late exponential phase cells of *A. baumannii* 17978 treated for 3 h. Untreated cells as the control (100% survival) were $2.3 \pm 0.4 \times 10^9$ CFU/ml, $4.8 \pm 2.5 \times 10^8$ CFU/ml and $2.1 \pm 0.3 \times 10^9$ CFU/ml, respectively. (d) Effect of colistin on the persister cells formation in the late exponential phase cells of *A. baumannii* MDR clinical strain MM6 treated for 3 h. Untreated cells as the control (100% survival) were $5.2 \pm 0.5 \times 10^9$ CFU/ml. The data is representative of three independent experiments. Bars represent the mean ± SD.

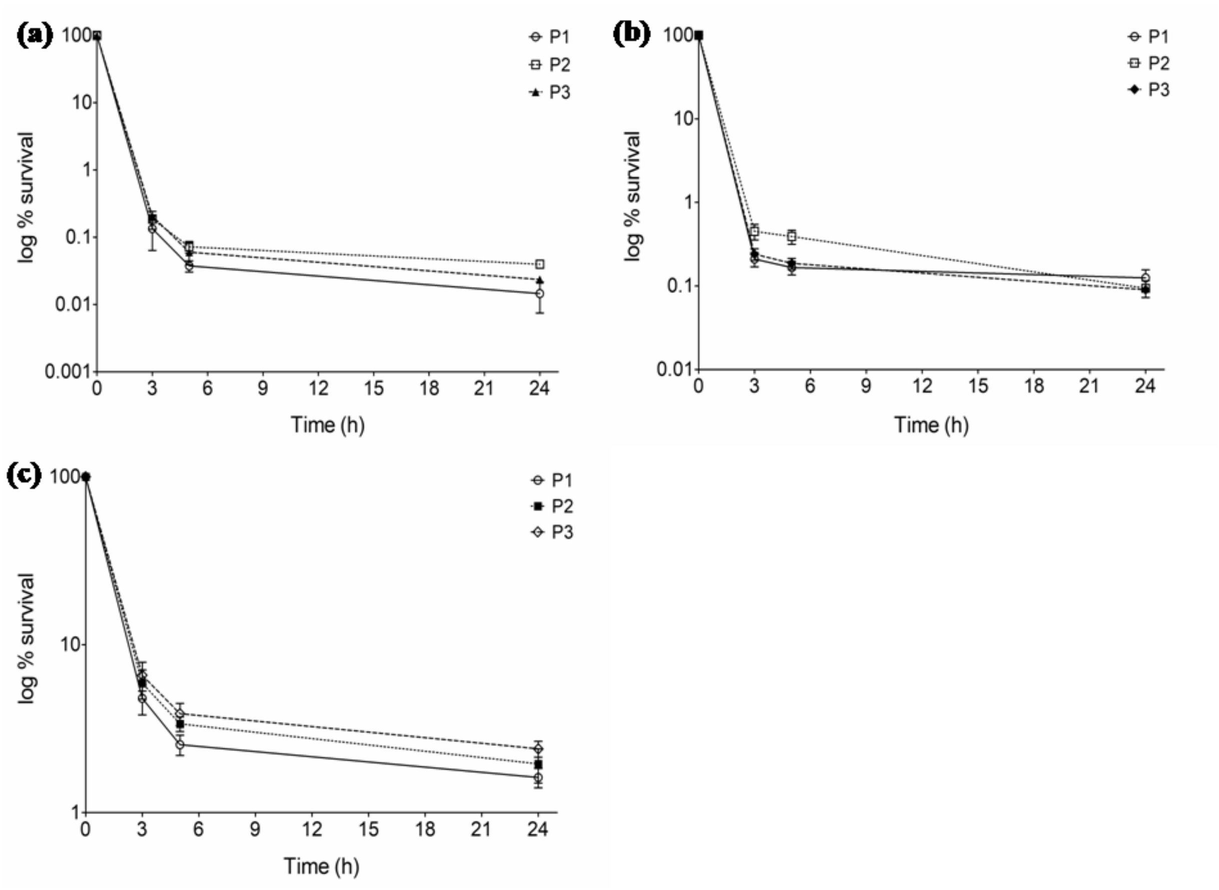

Suppl. Fig. 2: Non-heritability of the persister cells formed against antibiotics. An overnight culture of *A. baumannii* 17978 was inoculated in LB broth to obtain the late exponential phase cells and exposed to **(a)** colistin (10X), **(b)** amikacin (40X) and **(c)** rifampicin (20X) for 24 h. The surviving persister cells were regrown in antibiotic-free LB broth for 16 h and re-exposed to respective antibiotics for 24 h. The procedure was repeated for three consecutive passages (P1, P2 and P3). The data is representative of three independent experiments. Bars represent the mean ± SD.

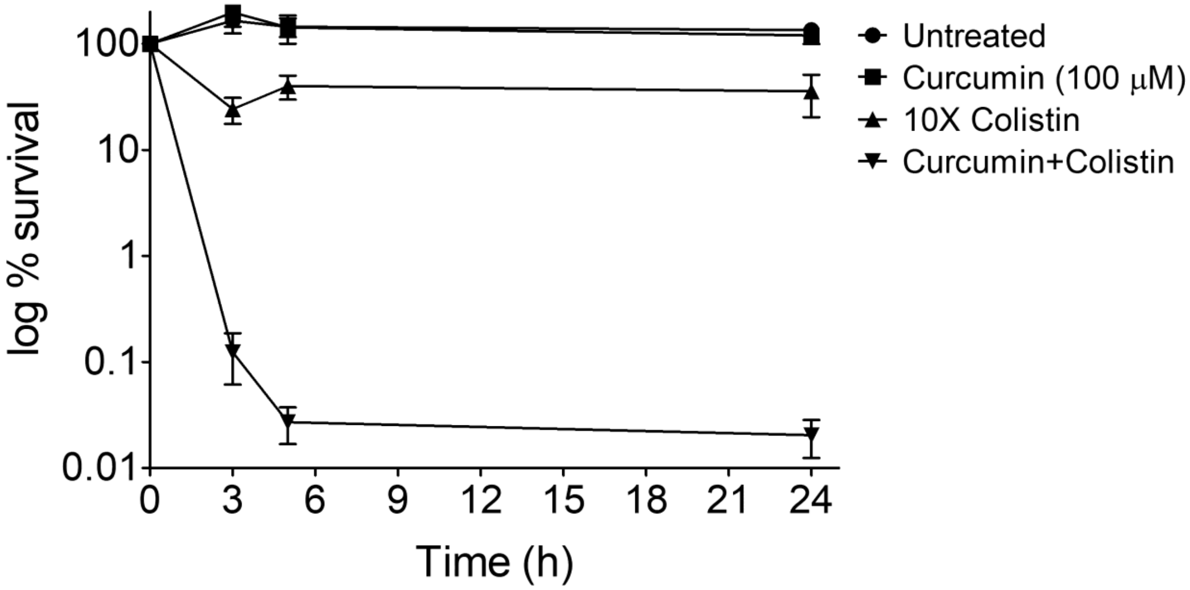

Suppl. Fig. 3: Effect of curcumin (100 µM) on the persistence of the late exponential phase cells of *A. baumannii* MDR clinical strain MM6 against 10X colistin. Untreated cells (100% survival) were 4.0±2.2 X 10^8 CFU/ml. The data is representative of three independent experiments. Bars represent the mean ± SD.

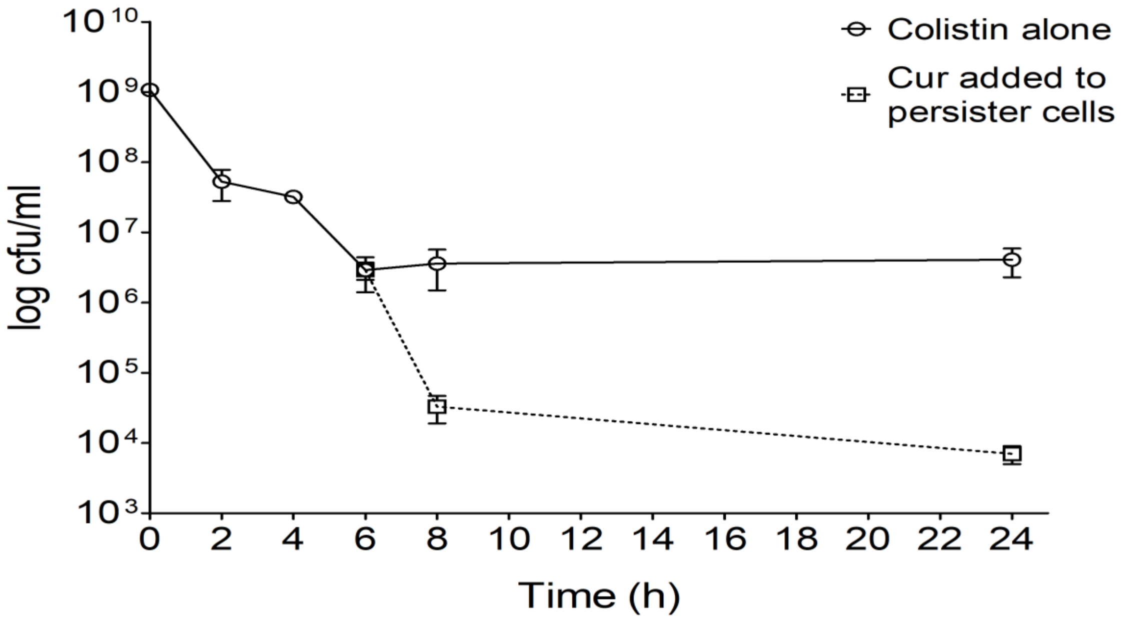

Suppl. Fig. 4: Effect of curcumin (100 µM) on the pre-formed persister cells against colistin (10X) in the late exponential phase cells of *A. baumannii* 17978. Curcumin (100 µM) was added to the persister cells obtained after treatment with 10X colistin for 6 h. The data is representative of three independent experiments. Bars represent the mean ± SD.

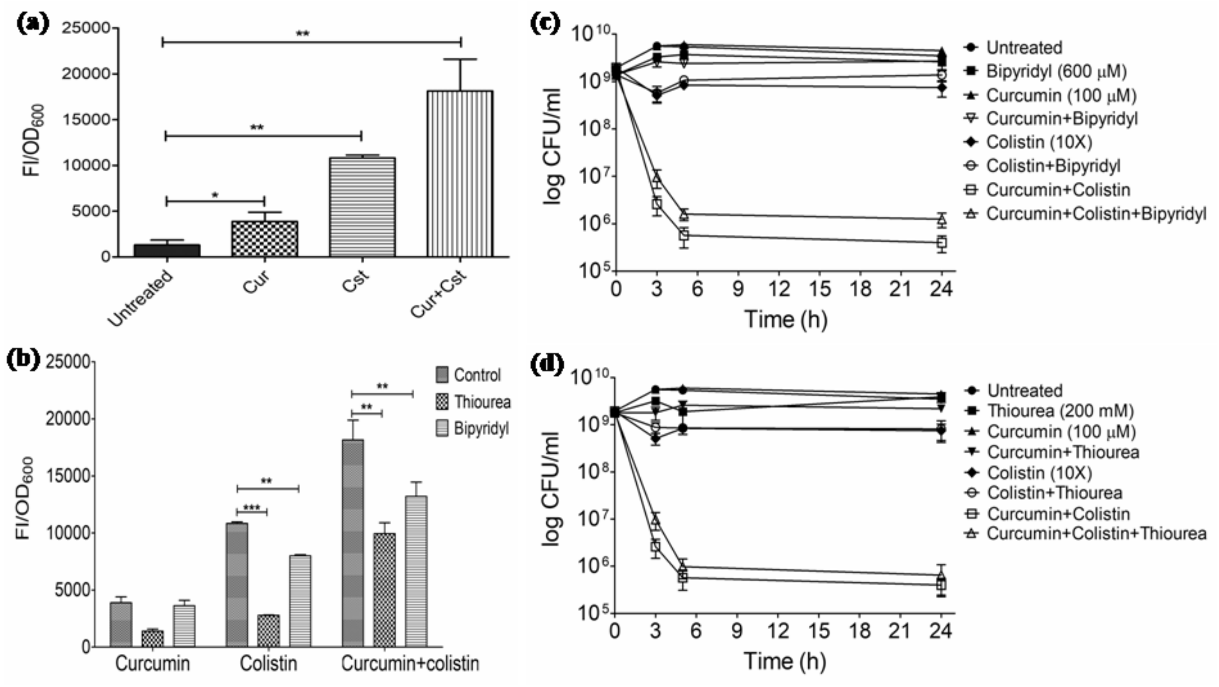

Suppl. Figure 5: Effect of ROS on the persistence of the late exponential phase cells of *A. baumannii* MDR clinical strain MM6. ROS levels in *A. baumannii* MM6 cells upon treatment with **(a)** 10X colistin (Cst) alone and in combination with curcumin (Cur; 100 µM); **(b)** curcumin (100 µM), 10X colistin alone and their combination, in presence of thiourea (200 mM) or bipyridyl (600 µM). Persister cells formation in the presence of **(c)** 2,2'-bipyridyl (600 µM), **(d)** thiourea (200 mM) against 10X colistin, 100 µM curcumin and their combination. CFU/ml for untreated cells (100% survival) for **(c)** 1.95±0.8 X 10^9 and **(d)** 1.4±0.6 X 10^9. The data is representative of three independent experiments. Bars represent the mean ± SD. *$P \leq 0.05$; **$P \leq 0.01$; ***$P \leq 0.001$.

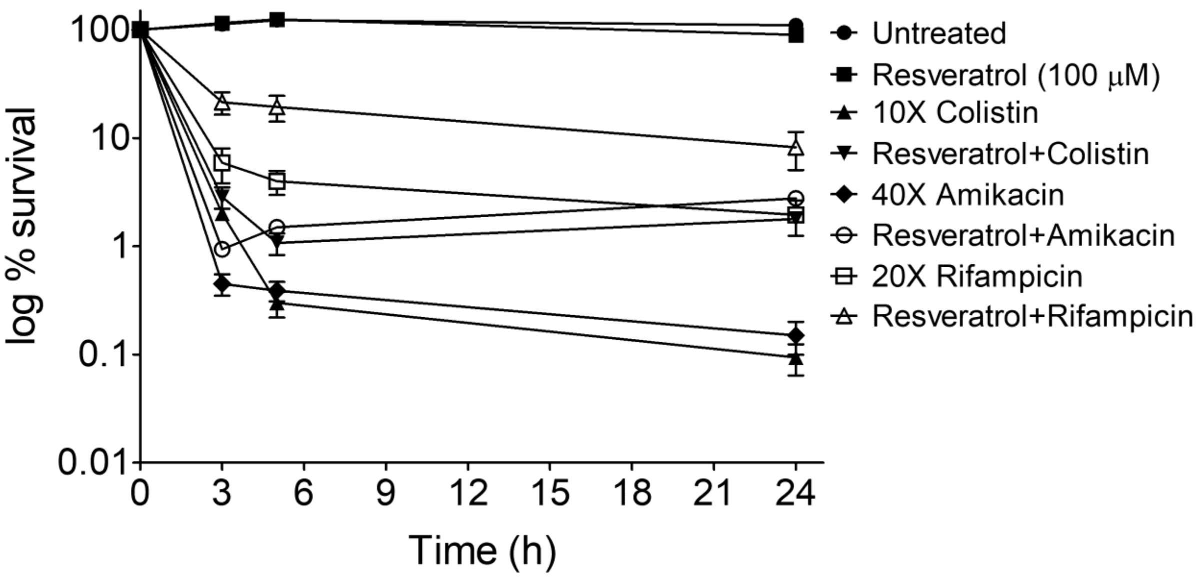

Suppl. Figure 6: Effect of resveratrol (100 µM) on the persistence of the late exponential phase cells of *A. baumannii* 17978 against 40X amikacin, 10X colistin and 20X rifampicin. Untreated cells and cells treated with resveratrol (100 µM) taken as the control (100% survival) were $9.0 \pm 0.4 \times 10^8$ and $8.0 \pm 1.2 \times 10^8$ CFU/ml, respectively. The data is representative of three independent experiments. Bars represent the mean ± SD.

Suppl. Table S1: Comparison of persister cells formation against amikacin, colistin and rifampicin in *A. baumannii* 17978 for 3 h at different growth phases.

Growth phase	Antibiotics	Percentage persister survival					
		**5X**	**10X**	**20X**	**40X**	**80X**	**100X**
**Mid exponential phase**	**Amikacin**	0.06	0.02	0.007	0.002	ND	ND
	**Colistin**	0.04	0.03	ND	ND	ND	ND
	**Rifampicin**	4.0	3.40	3.20	3.20	1.32	1.20
**Late exponential phase**	**Amikacin**	43.40	10.80	1.20	0.17	0.09	0.08
	**Colistin**	18.0	2.50	ND	ND	ND	ND
	**Rifampicin**	12.0	6.80	3.70	3.50	3.30	3.50
**Late stationary phase**	**Amikacin**	88.20	64.10	63.40	61.20	51.40	48.0
	**Colistin**	94.20	92.60	66.60	57.40	17.10	11.11
	**Rifampicin**	96.40	95.20	91.60	86.80	72.40	69.60

* ND: Not detectable.
